# Growth Retardation in Children with Kidney Disease

**DOI:** 10.1155/2013/970946

**Published:** 2013-09-25

**Authors:** Paulina Salas, Viola Pinto, Josefina Rodriguez, Maria Jose Zambrano, Veronica Mericq

**Affiliations:** ^1^Pediatric Nephrology Unit, Hospital Exequiel Gonzalez Cortes, Ramón Barros Luco 3301, Santiago, Chile; ^2^Faculty of Medicine, University of Chile, Av. Independencia 1027, Santiago, Chile; ^3^Faculty of Medicine, Catholic University, Av Libertador Bernardo O Higgins 340, Santiago, Chile; ^4^Institute of Maternal and Child Research, Faculty of Medicine, University of Chile, Casilla 226-3, Santiago, Chile

## Abstract

Growth failure is almost inextricably linked with chronic kidney disease (CKD) and end-stage renal disease (ESRD). Growth failure in CKD has been associated with both increased morbidity and mortality. Growth failure in the setting of kidney disease is multifactorial and is related to poor nutritional status as well as comorbidities, such as anemia, bone and mineral disorders, and alterations in hormonal responses, as well as to aspects of treatment such as steroid exposure. This review covers updated management of growth failure in these children including adequate nutrition, treatment of metabolic alterations, and early administration of recombinant human growth hormone (GH).

## 1. Introduction

Growth retardation remains a major problem in patients with chronic kidney disease (CKD). Initial reports of renal dwarfism date back to the turn of the twentieth century. Despite advances in conservative treatment and renal replacement therapies, 30–60% of patients with ESRD are short at adulthood [1].

The United States Renal Data System (USRDS) Pediatric Growth and Development Study showed that children with ESRD with moderate or severe growth retardation have higher rates of morbidity and mortality than children with normal growth rate [2].

This probably reflects poorer nutritional status and increased catabolism, which is associated with increased rates of infections, hospitalizations, and other adverse effects [3]. In addition, patients with short stature have an altered psychosocial development. Adult height of renal transplanted patients is statistically significantly (*P* < 0.001) related to marital status, the attained education, and employment level [3]. In patients with ESRD who have received some replacement therapy (dialysis or renal transplant) the risk of dying decreases by 14% for each height SDS increase [4]. 

Short stature is defined as a height below the 2 SDS for age and sex which corresponds to approximately the 2.5 percentile. It a statistical definition and thus ideally needs the use of ethnically appropriate growth charts [5]. In Chile, according to the National Registry of Chronic Kidney Disease of 2007, approximately 50% of patients with CKD have significant growth retardation. This growth delay is progressive, being −0.7 at birth (when they are affected since birth), −1.5 at start of dialysis, and −2.4 SDS at transplant. The 2006 Annual Report NAPRTCS (North American Pediatric Renal Trials and Collaborative Studies) reveals that children enrolled in the registry have about −1.61 and −1.78 mean height deficit for all patients at dialysis initiation and transplantation, respectively [6]. Children at the highest risk of short stature are affected infants (under one year) and patients with GFR less than 15% of normal. In fact, dialysis treatment for 6–12 months does not improve height SDS of these patients. However, patients transplanted during prepubertal years, particularly those under age 6, have a significant increase in growth rate after transplant [7]. 

Longitudinal bone growth is affected by intrinsic factors such as the life period. It is rapid in prenatal and early postnatal life but then slows with age and eventually ceases. This growth deceleration is caused primarily by a decrease in chondrocyte proliferation and is associated with other structural, functional, and molecular changes collectively termed growth plate senescence. Growth plate senescence results from a limited replicative capacity of the progenitor chondrocytes in the resting zone which is gradually exhausted [8]. During infancy a third of overall postnatal growth occurs; therefore, circumstances affecting the growth rate at this stage will cause severe growth retardation and probably irreversible loss of growth potential. 

Recent experimental findings from laboratory and clinical studies suggest that growth plate senescence explains the phenomenon of catch-up growth. Growth-inhibiting conditions delay the program of growth plate senescence. Consequently, growth plates are less senescent after these conditions resolve and therefore grow more rapidly than normal for age, resulting in catch-up growth [8].

It has been suggested that the growth retardation of fetuses and infants is influenced by nutritional and metabolic alterations. In infants with CKD, malnutrition is secondary to poor intake, frequent vomiting, catabolic processes, renal losses of water and electrolytes, renal osteodystrophy, anemia, and metabolic acidosis [1, 9]. All these factors contribute to inadequate linear growth. 

Most infants with CKD need supplementary feeding to maintain adequate water, electrolytes and nutrient requirements [1]. In this regard, patients with CKD in the predialysis stage, supported with early nutritional intervention to prevent metabolic disorders, may achieve adequate growth rates as good as 22.2 cm during the first year, 10.9 cm during the second, and 7.6 cm during the third year of life [9].

During childhood, growth rate in CKD patients is mostly correlated with glomerular filtration rate and patients maintain an adequate growth pattern if their GFR is greater than 25 mL/min/1.73 m^2^ [1]. However, the observed growth rate should be evaluated periodically, as there are reports of stunting at all levels of renal dysfunction. During this period of life growth rate is primarily determined by an appropriate secretion and action of growth (GH) and thyroid hormones, and a normal expected growth rate should be within 5–7 cm/year [9]. During puberty, appropriate growth spurt is also directly determined by sex steroids which also affect GH secretion [10]. Both will determine linear growth and in addition the development of secondary characteristics and fertility. 

It has been observed that patients with ESRD have a 2-year delay in the onset of clinical pubertal changes and a delay of approximately 2.5 years in attaining their growth spurt. Furthermore, in patients with CKD, the growth spurt is of shorter duration and intensity, causing height gain in this period to be reduced by 45% compared to healthy children. This pubertal delay is also seen in kidney transplant patients. It is also important to realize that in patients on dialysis, despite reaching pubertal maturity, a significant number of them have disorders of reproductive function [9, 11].

The most important determinant factors on adult height of patients with ESRD are the etiology of the disease, age of onset, and duration of CKD [12]. 

## 2. Pathogenesis of Growth Delay in CKD

Growth retardation in CKD is multifactorial and influenced by the degree of renal dysfunction, but also by comorbidities, factors associated with the treatment modality, and the genetic growth potential determined by midparental height [13]. 

### 2.1. Underlying Disease

Kidney diseases have very different pathogenesis which will influence growth pattern. In congenital diseases which present early in life with CKD, growth retardation is more significant than in acquired CKD. Renal dysplasia is associated with significant water and electrolytes losses, which must be supplemented to enable proper growth. Glomerular diseases present with early growth retardation even in early stages of CKD, mainly due to the nephrotic state and steroid treatment. In the case of tubular dysfunction which presents with electrolyte, bicarbonate, and water loss, growth failure occurs even with normal glomerular function. Among tubular diseases, the most severe growth retardation occurs in Fanconi syndrome [13].

### 2.2. Protein-Calorie Malnutrition

Infants and young children have high energy demands and low levels of energy stores, and therefore, are especially vulnerable to nutritional imbalances which will prevent a normal growth velocity. In children with CKD, several factors contribute to malnutrition: progressive anorexia, decreased protein synthesis, and increased catabolism. It should be noted that malnutrition is an important factor to consider because it is associated with significantly increased mortality [2, 4]. Patients with CKD should receive at least 80% of the age-appropriate dietary recommendations to achieve a normal growth rate. However, increases above the recommended dietary intakes produce no further increase in growth but lead to obesity. 

### 2.3. Metabolic Acidosis

Defects in acid-base homeostasis are frequently observed in patients with CKD. Generally this becomes evident when GFR is lower than 50% of normal. Metabolic acidosis is influenced by protein intake and acid load, protein catabolism, and electrolyte abnormalities. There is a significant relationship between protein catabolism (measured by the presence of leucine) and decreased linear growth [14].

Experimental studies have shown that, with metabolic acidosis, uremic animals have increased glucocorticoid production, increased protein degradation, decreased spontaneous GH secretion from the pituitary, and in addition GH insensitivity due at least in part to decreased GH receptor expression, decreased circulating IGF-I concentrations, and decreased IGF-I receptor expression [9].

### 2.4. Disorders of Water and Electrolyte Metabolism

In CKD patients, it has not been formally proven that the ratio of water and electrolyte loss is associated with growth retardation. However, there is clinical evidence showing that patients with salt-losing tubulopathies have severe growth retardation, which is partially resolved with appropriate electrolyte supplements. In turn, growth retardation is a common finding in patients with uncontrolled diabetes insipidus. 

### 2.5. Metabolic Bone Disease

Patients with CKD have severe alterations in the PTH-calcium-phosphorus metabolism, which can lead to growth retardation. Secondary hyperparathyroidism with excessive levels of PTH interferes with longitudinal growth, as it promotes the destruction of the growth plate architecture, with more frequent slipped capital femoral epiphysis and metaphyseal fractures. However, small doses of PTH are required to stimulate the expression of vitamin D receptor in the growth plate and also to stimulate local synthesis of IGF-1 which will stimulate local proliferation and differentiation of the growth plate chondrocytes [15].

In patients with CKD, very low levels of PTH cause low-bone turnover disease and severe stunting. In summary, the level of PTH must be balanced, to prevent a high- or low-bone turnover disease. PTH values range from 110 pg/mL in CKD I-IV to 250 pg/mL in ESRD [16]. 

### 2.6. Anemia

Classically, chronic anemia has been correlated with growth retardation and delayed psychomotor development. These consequences may be due to different mechanisms, such as poor appetite, recurrent infections, cardiac complications, and reduced oxygen supply to different tissues including the cartilage. The correction of anemia in patients with CKD by using human recombinant erythropoietin has improved exercise capacity, decreased heart rate, and oxygen consumption at rest [17]. Nevertheless, multicenter studies have observed no persistent catchup in longitudinal growth. 

### 2.7. Alterations in the Gonadotropic Axis

In patients with CKD, abnormalities at different levels of the gonadotropic axis are observed, influencing the sexual and pubertal delay in these patients. At the hypothalamic level, experimental evidence suggests that there is a decreased secretion of GnRH [18]. This inhibition may be mediated by increased availability of the inhibitory neurotransmitter aminobutyric acid [18]. On the other hand, LH concentrations are elevated due to a decreased kidney clearance. However, the pulsatile nocturnal secretion of LH which is finally responsible for onset of puberty is decreased by 70%, related to altered GnRH release [19]. Once puberty is started, ESRD males generally show decreased free testosterone and dihydrotestosterone levels. There is a decrease of the activity of 5 a reductase which metabolizes testosterone into the most active form of dihydrotestosterone. In addition there is lower clearance of sex hormone-binding protein (SHBG) [20], thus decreasing free testosterone. Finally, the plasma concentration of inhibin is elevated [21]. In females estradiol levels tend to decrease in proportion to the decrease in GFR [22].

In summary, insufficient GnRH secretion at the hypothalamic level, reduced nocturnal LH pulsatility, and decrease in gonadal hormones are the basis for the pubertal delay observed in patients with CKD. 

### 2.8. Disorders of Somatotrophic Axis

GH is the key hormone responsible for postnatal growth. The anterior pituitary secretes GH in a pulsatile fashion stimulated by GHRH and inhibited by somatostatin. Growth hormone exerts its action primarily through IGF-1. Recently, ghrelin, a 28 aa peptide, secreted in the stomach fundus and in lesser amounts at the hypothalamus and pancreas, has been found to also participate in the control of GH release. Ghrelin levels increase with fasting, cholinergic stimulation, IGF-I, and estrogens and decrease with food intake. The role of this hormone in the control of GH secretion and action in CKD has not yet been defined [9, 23]. In patients with CKD GH secretion varies considerably. In prepubertal patients endogenous GH secretion is normal or elevated, due to the attenuated IGF-I negative feedback [24]. In adolescent patients, however, GH secretion is diminished, probably related to the decrease of the gonadal hormones which usually enhance GH secretion. GH secretion is also influenced by changes in nutritional intake and the degree of metabolic acidosis [9]. GH clearance at the kidney decreases as the GFR decreases, leading to a 50% decrease in patients with ESRD. 

GH-binding protein (GHBP) and the density of GH receptors in the liver and also at the growth plate are decreased, which results in resistance to GH action. This resistance is proportional to the degree of renal dysfunction [9]. At the postreceptor level, GH signals through Januskinase (JK)/signal transduction and activation of transcription (STAT) factors. GH binds to its receptor which activates JAK-2 which in turns stimulates tyrosine kinase to phosphorylate STAT1, STAT3, and STAT5. Once these are phosphorylated, they form dimers which enter the nucleus, activating genes that produce IGF-I and genes that produce inhibitory cytokines (SOCSs). SOCS dephosphorylate STAT, causing a negative feedback action of GH [25]. 

**Figure 1:**
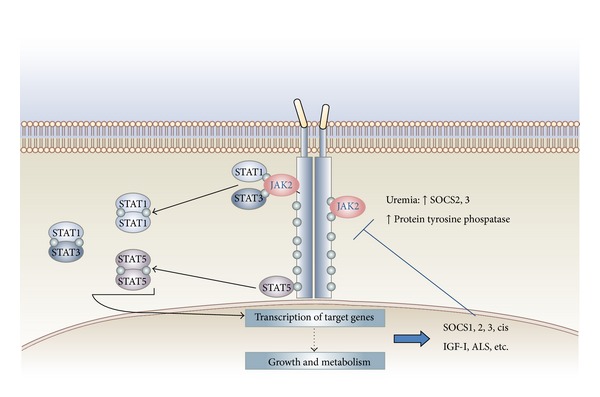
Growth hormone resistance in uremia: role for impaired JAK/STAT signaling.

When uremia is present, defects in GH-JAK2 activation, in STAT transduction, and also in exaggerated expression of SOCS2 and SOCS3 are observed, causing suppression in GH signal transduction. These alterations are increased when inflammatory cytokines are present in situations of chronic uremia [25, 26] (Figure 1).

Several studies have shown that longitudinal growth is mediated in part by stimulation by IGF-I at the growth plate. Classically, IGF-I was assumed to be provided by the liver and through the blood would target the bone epiphyseal growth plate. However, it is now recognized that an important source of IGF-I is that produced locally in a paracrine fashion in response to circulating GH.

In children with CKD, growth has been linked to the free IGF-I levels [13]. IGF-I circulates in serum as a 150 kD complex trimer which includes IGF-I, IGF binding protein (IGFBP), and acid labile subunit (ALS). In CKD patients a decreased renal clearance of IGFBP is observed, which leads to an excess of IGFBP-1 in the circulation and the concomitant decrease in the levels of bioactive IGF-I. The decrease is correlated with the degree of renal failure [9]. 

**Figure 2:**
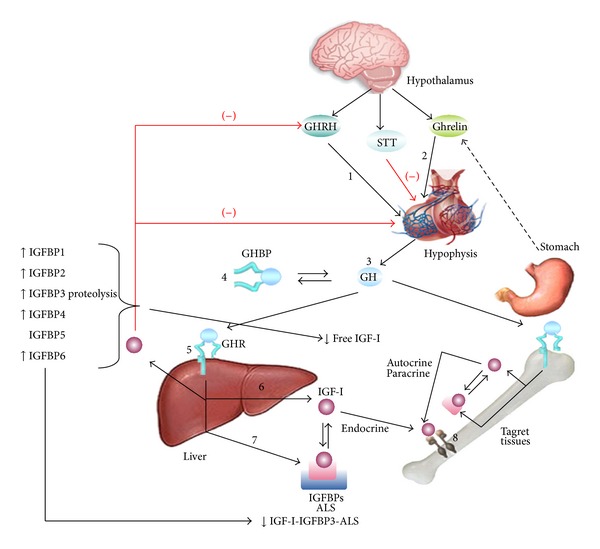
GH-IGF-I physiology. Several abnormalities in the GH and IGF-I axis in pediatric patients with CKD. IGF-I resistance likely reflects an increase in circulating IGFBP-1, -2, -4, and -6, which lead to a reduction in bioavailability IGF-I. In addition, increased IGFBP3 proteolysis leads to a reduction in the IGF-I-IGFBP3-ALS complex. The reduced IGF-I bioavailability and increased concentration of IGFBP-1 and -2 in patients with CKD are thought to contribute to the lack of GH responsiveness. In addition the direct GH effects on bone are inhibited.

Several studies in CKD have demonstrated increased IGFBP-1, -2, -4, and -6 and increased fragmented IGFBP-5 and IGFBP-3 (the latter in the form of low molecular weight), with high affinity to IGF-I [25]. In healthy children there is an excess of 25% of IGFBP over IGF-I, in CKD children this ratio is 150%, and in ERCT the excess is 200% over the IGF, while significantly reducing IGF-I bioavailability [9, 27]. This IGFBP increase contributes to the growth retardation and to the reduced response to GH treatment [9]. An increase in IGFBP-1, -2, -6 inhibits IGF-dependent growth of chondrocytes. Thus increases in IGFBP-1 and -2 have been negatively correlated with longitudinal growth in patients with CKD [28] (Figure 2). 

Finally, it is known that IGF-I signals through the IGF-I receptor (IGF-1R), binding first the *α* subunit of the IGF-I receptor, activating the tyrosine kinase of the *β* subunit, and initiating the downstream signal. In experimental studies in uremic rats, a defect in the postreceptor activation pathways of IGF-I has been reported. This defect could also affect the strength of IGF-I in skeletal muscle metabolism in patients with CKD [9, 29].

### 2.9. Steroidal Treatment

CKD patients receive steroid treatment, either for glomerular disorders or as part of posttransplant immunosuppressive therapy. Steroids can reduce longitudinal growth at various levels of the somatotropic hormone axis [30]:

- decreased pulsatile GH secretion from pituitary,
- reduced physiological GH increase during puberty, mainly by increasing hypothalamic somatostatin,
- postreceptor insensitivity to GH and IGF-1 at the level of the liver and at the growth plate,
- decreased paracrine synthesis of IGF-1 and interference with synthesis of IGFBP.

Steroids also affect calcium/phosphorus metabolism which also affect growth. Steroids are associated with increased incidence of fractures, reduced bone density, decreased activity of 1 *α* hydroxylase thus decreasing 1.25 OH vitamin D, increased calciuresis, and avascular necrosis.

## 3. Treatment of Growth Failure in CKD

Current challenge in the clinical management of pediatric patients with growth failure is to optimize all aspects of treatment, including correction of metabolic disorders and appropriate use of hormone therapy. In CKD data from the NAPRTCS 2008, from 668 enrolled patients with short stature and Tanner pubertal stage less than IV, only 57.7% of them had a CO_2_ >20 mEq/L and PTH concentration below twice the normal level. Furthermore, only 30.5% were treated with GH. These data emphasize that medical treatment is not optimal in most children with CKD [13]. 

Herein we present nutritional and metabolic interventions to consider prior to starting GH therapy.

### 3.1. Nutrition

In infants and young children with CKD, the most important action to prevent growth retardation is to achieve an adequate caloric intake. For this goal, it is usually required to supplement feeding through nasogastric tube or gastrostomy. In older children, it is important to provide an appropriate nutrition although, when using this as the only intervention, patients do not reach catch-up growth [31]. It is recommended that the caloric intake should reach 80−100% of the daily recommended allowance (RDA) for healthy children. An intake over 100% of the recommendations does not improve growth and promotes obesity. 

With regards to protein intake, patients should receive the daily protein recommendations for age. Interestingly, in patients receiving peritoneal dialysis therapy, an increase of 0.2 g/kg/day above those recommendations is indicated, to compensate for losses through the peritoneum. Higher protein intakes are harmful because they promote metabolic acidosis and phosphorus overload. 

### 3.2. Metabolic Acidosis

In patients with CKD, the exact level of acidosis correction to achieve maximum growth has not been determined. Empirically NKF K/DOKI guidelines are used, which suggest treating with alkali, to reach levels of HCO_3_ about 22−24 mEq/L [13]. 

### 3.3. Supplements

To allow maximum growth potential, supplements must be delivered according to the etiology of CKD; in salt-losing renal diseases associated with polyuria supplementation of water and electrolytes is required [32]. 

### 3.4. Management of Metabolic Bone Disease

Calcitriol deficiency produces secondary hyperparathyroidism and renal osteodystrophy; therefore, a low phosphorus diet together with phosphate binders and vitamin D analogs is indicated with the aim of halting the PTH secretion. This suppression must be controlled to avoid falling into a low-bone turnover disease and/or a dynamic bone disease. The optimal level of PTH to achieve maximum growth in children with CKD has not been delineated, but it is suggested that in ESRD stage IV and V a value approximately 3 times the upper limit of normal is appropriate. In ESRD I, II, or III the PTH value should reach the upper limit of normal [13]. As recommended by NKF K/DOQI should be an intact PTH below 500 pg/mL [33] and phosphemia must be in a range of 1.5 times the normal value for age. 

### 3.5. Influence of Dialytic Therapies in Height of Patients with CKD

Dialysis partially corrects the uremic syndrome, but clearance through dialysis fails to improve the growth retardation. Only the maintenance of a residual renal function allows a better longitudinal pattern through specific renal clearance. The permeability of the peritoneal membrane used for peritoneal dialysis, as a dynamic dialysis membrane with an exchange surface area recruitment capacity and unique permeability characteristics, also plays a role with regards to growth impairment. High peritoneal transporter status is associated with subnormal longitudinal growth in children on chronic peritoneal dialysis [34]. In adults, high transporters are associated with higher morbidity and mortality, probably secondary to a condition of persistent microinflammation. This microinflammation state may produce GH resistance. A pediatric study showed that higher peritoneal transport rate was associated with mortality only in a univariate analysis. However, the multivariate analysis showed that the association with mortality was only with low serum albumin [34]. Fishbach and Warady reported that, in children receiving peritoneal dialysis, impaired growth was associated with adequate urea Kt/v (urea) and low creatinine clearance [35].

Related to children in ESRD receiving hemodialysis, recent studies have shown that longer hemodialysis or daily dialysis could induce catch-up growth [34–36].

### 3.6. Influence of Transplantation on the Size of Patients with CKD

Growth rate after ESRD in children who have undergone a kidney transplant varies widely, from those children who have a catch-up growth that allows them to reach a normal height, and those children who end up as short statured adults. The most important factors to achieve an adequate adult height are an appropriate glomerular filtration rate, transplantation at an early age of life, the beginning of the pubertal growth spurt at an appropriate time, and posttransplant immunosuppression without steroids or early steroid withdrawal [30].

The decline in graft function significantly impacts growth rate. Tejani and Sullivan (1993) showed that, for every 1 mg/dL of creatinine rise, height decreases by −0.17 SDS [37]. 

Regarding the age when transplant is performed, earlier transplant is associated with a more-intense catch-up growth, and in addition allows the patient to achieve a better pubertal spurt. Patients who are transplanted during adolescence show a suboptimal pubertal spurt; therefore, it has been suggested that they should undergo GH treatment in an attempt to maximize the peak pubertal spurt [13]. 

For many years steroids have been the cornerstone of immunosuppressive therapy after pediatric renal transplant. However due to the multiple adverse effects, in recent decades there has been an attempt to minimize or even avoid the use of steroids in immunosuppressive protocols. According to the Kidney Disease Improving Global Outcomes (KDIGO) 2008, steroids may be withdrawn the first week after transplantation, which helps to minimize adverse effects without affecting graft survival. Candidate patients for this procedure are those of low immunological risk and need to have received an induction therapy [38]. Grenda recently performed a metaanalysis of available studies of steroid minimizing or steroid withdrawal in pediatric transplantation immunosuppressive protocols. The author concluded that steroid-free protocols and early withdrawal of steroids are safe and effective in renal transplant recipients of low immunological risk [30]. 

In Chile, Delucchi et al. performed a protocol of early steroid withdrawal in low immunological risk patients showing benefits in growth rate for 5 yrs after transplant in prepubertal and pubertal patients [39]. In addition, we recently showed that, aside from growth benefits, early steroid withdrawal improved lipid profile and the percentage of fat in the trunk and extremities in prepubertal patients with kidney transplants [40]. 

### 3.7. Using GH

There is ample evidence that the use of recombinant human growth hormone (rhGH) is an effective and safe therapy for the treatment of growth failure in CKD patients. GH administration significantly increases production of IGF-I in the liver and growth plate and only slightly increases the IGFBP synthesis, thus allowing normalization of the IGF-I bioactivity, improving longitudinal growth to achieve a normal adult height in children with CKD. Furthermore, anabolic effects are also observed with GH use: weight gain and increased muscle-mediated middle arm circumference, which are also beneficial for these patients [9]. 

In prepubertal patients, GH response depends mainly on the child age, residual GFR, growth rate prior to the start of treatment, and midparental height. The best response to GH is observed in patients in predialysis stages, probably due to a better GH sensitivity. Infants who do not have appropriate growth, in spite of adequate caloric intake, also benefit from hormonal therapy [1].

During the onset of puberty, prior to growth spurt, a flattening of growth rate is often observed. However GH treatment should be maintained during this period since subsequently GH produces an increase in growth rate that is higher in patients who have continued treatment with GH [41].

In summary, the cumulative height gain during the treatment with GH is positively affected by the duration of GH treatment and negatively by a shorter height at the start of GH treatment and the time of dialysis [12]. Sixty-five percent of CKD patients treated with GH reach an adult height within the normal range. Nevertheless, adult height achieved is significantly less than their genetic potential [12].

In renal transplanted patients, even with immunosuppressive therapy including steroids, GH therapy has shown to induce a significant increase in growth rate when used from the prepubertal age until reaching adult height. In these patients, catch-up growth is associated with increased levels of IGF-I [27]. 

### 3.8. Future Therapies

Since in CKD growth failure is not caused by GH deficiency, but rather a state of GH resistance, new treatments modalities aimed at minimizing this defect are being explored. These therapies are based on recombinant IGF-I (rhIGF-I), recombinant IGF-binding proteins (rhIGFBP3), and IGF-I displacers. They are IGF-I analogs, which have an affinity for IGFBP but have no effect on IGF receptors, thus “displacing” IGF-I from IGFBP and elevating free IGF-I levels [9, 42]. RhIGF-I has been used in patients with GH resistance due to GH receptor mutations, and a modest increase in growth rate has been observed, although lower than the results demonstrated with GH in growth hormone-deficient conditions. RhIGF-I therapy also induces an increase in GFR and renal plasma blood flow in patients with ESRD and in healthy subjects [9, 43, 44]. The use of rhIGF-I is associated with side effects by increasing bioactive IGF-I, and GH axis suppression, with a decrease in IGFBP3-ALS synthesis, and hypoglycemia by increased rhIGF-I binding to insulin receptors. To reduce these effects, it has been proposed to use the combination of rhIGFBP3 rhIGF-I [41]. Animal studies have used a combination of rhGH + rhIGF-I, achieving an additive effect on longitudinal growth and anabolism [9]. 

Finally, two IGF-I analogs have been studied which allow the release of IGF-I from binding protein, increasing free IGF-I. Experimental studies have shown that rats undergoing this therapy have renal enlargement, increased renal and bone growth. The effect is additive when combined with rhIGF-I [9].

## Abbreviations

CKD: Chronic kidney disease
ESRD: End stage renal disease
NAPRTCS: North American Pediatric Renal Trials and Collaborative Studies
SDS: Standard deviation score
GFR: Glomerular filtration rate
KDIGO: Kidney Disease Improving Global Outcomes.
